# Providing choice enhances reading motivation

**DOI:** 10.1177/17470218251370916

**Published:** 2025-08-18

**Authors:** Amrita Bains, Carina Spaulding, Jessie Ricketts, Saloni Krishnan

**Affiliations:** 1 Department of Psychology, Royal Holloway, University of London, Egham Hill, Surrey, UK; 2 Department of Experimental Psychology, University of Oxford, Oxford, UK; 3 The Reading Agency, London, UK; 4 Division of Psychology and Language Sciences, University College London, London, UK

**Keywords:** Reading, motivation, intrinsic reward, decision making, choice, agency, autonomy

## Abstract

Multiple literacy programmes embed a choice of reading material into their programmes, as this is believed to enhance motivation for reading. Yet, this practice has not been experimentally evaluated. Is choice effective at boosting reading motivation? Is the nature of choice provided important? Using a new experimental paradigm to tap reading motivation, we assessed whether reading enjoyment and willingness to pay for books were influenced by having: (a) a choice of book; or (b) a choice of book genre. Having choice increased both reading enjoyment and the amount participants were willing to pay for books. Our results show that choice boosts enjoyment for reading. This has implications for the design of literacy programmes, indicating that incorporating choice in such programmes is beneficial.

## Introduction

Choice is argued to be a crucial driver of reading motivation, comprehension, and reading engagement (Guthrie & Humenick, 2004; Guthrie, Wigfield, et al., 2004; Patall et al., 2008; Wigfield et al., 2008). Providing and supporting reader choice is a central emphasis in reading programmes and interventions, as well as in teacher training (Brandt et al., 2021; McGeown & Wilkinson, 2021; McGeown et al., 2023). Examples of programmes which incorporate the choice of reading material as an important practice include the Reading Agency’s Summer Reading Challenge, the Whole School Reading Project by the Give a Book charity, the Nuffield Love to Read programme (McGeown et al., 2023), the Young Readers programme, the BookBuzz programme by BookTrust, and the World of Stories programme by the National Literacy Trust. Somewhat surprisingly, very few studies have directly evaluated whether reader choice influences reading motivation. Such evaluation is critical for developing a better understanding of the mechanisms that drive motivation, as well as understanding the most effective ways of providing choice. We recently developed an experimental paradigm to gauge the value readers place on text, assessing whether they are willing to incur temporal or monetary costs to gain more information about books (Bains et al., 2023). In this study, we adapt our new experimental paradigm to capture how different kinds of choice affect aspects of motivation, such as enjoyment and willingness to pay for books.

### Why does providing choice enhance motivation?

Providing choice over texts and tasks is postulated to increase reading motivation and engagement as it allows readers to exercise personal control, expressing their preferences and ideas (McGeown et al. 2023; Moley et al. 2011). Indeed, according to the Self-Determination Theory, intrinsic motivation increases when people can make choices which align with their values, as this fosters a sense of autonomy (Diefendorff & Seaton, 2015; Ryan & Deci, 2000; Ryan et al., 2021). Here, autonomy is considered a sense of initiative and ownership in one’s actions, which is supported by experiences of interest and value and undermined by experiences of being externally controlled, whether by rewards or punishments (Ryan & Deci, 2020). The authors have suggested that autonomy, alongside competence and relatedness, is a basic psychological need, and children require environments that can support these needs (Ryan & Deci, 2020). While the provision of choice can boost autonomy, meaningless choices such as two options that are irrelevant to the reader are less important for autonomy (Assor et al., 2002; Ryan & Deci, 2020). Educational theories, therefore, largely emphasise that the *content* of a choice is intrinsically linked to motivation, suggesting that books must be aligned with reader interests and identity (McGeown et al. 2023; Wigfield & Guthrie, 1997). There is evidence that when students read books they are interested in, they are willing to spend more time and effort on learning the material they encounter (Jones et al., 2025; Turner & Paris, 1995), and that providing opportunities for such choices can boost both motivation and comprehension (Guthrie et al., 2006). Moreover, studies with young adolescents have shown that students identify choice and personal relevance as key motivators for reading (Ivey & Broaddus, 2001), reinforcing the view that fostering autonomy through meaningful reading choices can enhance both motivation and learning outcomes.

A complementary body of research from cognitive psychology and neuroscience suggests that choice not only reflects existing preferences but also actively shapes them. In adult studies, choice has been shown to increase the desirability of a chosen stimulus (Brehm, 1956; Egan et al., 2007; Lieberman et al., 2001; Sharot et al., 2010). For example, Sharot et al. (2009, 2010) found that people rated holiday destinations they chose as more desirable than the destination they rejected in a choice task. This was even when they rated both destinations as similarly desirable prior to making the choice. In a similar vein, adults were more likely to wait to learn the outcome of a lottery they chose to play compared to a preselected lottery (Verdugo et al., 2022). This shows that the sense of agency provided by choice is highly valued, even when controlling for interest in the stimulus. Neuroimaging studies further support this idea, revealing that actively chosen rewards elicit stronger activation in reward-related brain regions than passively received rewards (Goulet-Kennedy et al., 2016; Tricomi et al., 2004). Notably, even anticipation of personal control or the opportunity to choose engages these neural circuits (Leotti & Delgado, 2011; Leotti et al., 2010).

Together, these two perspectives suggest that allowing children to choose what they read may not only align with their existing interests, but also actively shape and deepen those interests over time. In this way, providing choice could both reflect and cultivate engagement, making reading a more meaningful and self-reinforcing experience.

### Experimental evaluations of the efficacy of choice

Researchers have examined whether the extent to which school and home contexts are supportive of children’s autonomy predicts motivation. In these studies, children typically rate whether they have opportunities for autonomy in the classroom, for example, rating 1 to 5 in response to questions like ‘My teacher encourages me to ask questions’. A recent meta-analysis of educational studies indicates that perceived teacher and parent autonomy support increases motivation (Bureau et al., 2022). A large longitudinal study revealed that when children perceived greater support for autonomy from teachers, they showed higher levels of intrinsic motivation (Engler & Westphal, 2024). Choice has also been evaluated within a programme evaluation of Concept-Oriented Reading Instruction (CORI), where it is a key instructional practice. In CORI, teachers allow children to generate questions about a topic chosen by the children, and select their own reading material for a project from a book list. CORI’s efficacy was tested in 120 children aged 8 to 9 (Guthrie & Humenick, 2004; Guthrie, Wigfield, et al., 2004; Guthrie et al., 2007; Wigfield et al., 2008). Students reported higher motivation for reading when using CORI and increased interest in the books they were selecting (Guthrie & Humenick, 2004; Guthrie, Wigfield, et al., 2004; Guthrie et al., 2007; Wigfield et al., 2008). These studies strongly suggest that providing readers with choice has important long-term effects on motivation. Yet, given their naturalistic settings, these studies have not specifically isolated or manipulated choice. Therefore, factors beyond choice alone, such as responder bias or evocation of certain reading environments by motivated readers, might explain these results.

Only three experimental studies have investigated the influence of providing choice on reading activities (Flowerday et al., 2004; Fridkin, 2018; Marinak & Gambrell, 2008). Flowerday et al. (2004) gave college students a choice between reading and answering questions from two sets, A or B. They found that providing choice did not have a significant effect on learning or engagement. In contrast, unpublished research by Fridkin (2018) revealed that when 8- to 9-year-old children were given a choice of passage to read, they had higher comprehension accuracy, relative to the group given no choice. The last study examined the effect of providing a choice of reward. Students who were given the choice of a book as a reward, rather than a token, were more likely to engage in subsequent reading (Marinak & Gambrell, 2008). Critically, all these experimental studies have used between-subject designs where participants make a single choice, limiting our ability to draw strong conclusions about the influence of choice.

### Rationale

Drawing on the decision science literature, we have developed an experimental paradigm to measure situational interest in reading (Bains et al., 2023; Jones et al., 2025). This paradigm provides us a robust tool for investigating contextual or situational factors that can drive motivation. In this paradigm, we obtain two dependent measures of motivation, drawing on neurobiological literature that suggests that motivation is not a unitary construct, but a collection of similar but differentiated cognitive processes including ‘liking’, ‘wanting’ and ‘learning’ (Berridge & Kringelbach, 2008). Enjoyment of a text offers us a measure of ‘liking’, while the amount someone is willing to pay offers a measure of ‘wanting’. This is because the cost a participant is willing to incur, which could be monetary (Becker et al., 1964), temporal (Frederick et al., 2002; Hayden et al., 2007) or effort (Le Heron et al., 2018; Westbrook & Braver, 2015), can act as a proxy for the subjective value or desirability of the stimulus. Here, we adapt this paradigm to measure the influence of different choices on reading enjoyment and willingness to pay for a book. We hypothesised that having a choice would be associated with increased enjoyment of a book. Further, we explored whether choice would also enhance the willingness to pay more. We conducted two within-subject experiments, contrasting trials in which participants could choose between two options compared to trials in which a computer made the choice, allowing us to assess the influence of choice on these two ratings. In Experiment 1, we tested whether providing a choice of book would influence enjoyment and willingness to pay. In Experiment 2, we investigated whether the same effects would hold when adults were given a choice of genre.

## Experiment 1: providing choice within genre

In this within-subjects experiment, we assessed whether having choice influenced reading enjoyment and willingness to pay for books. Participants made 32 decisions: they were shown two books drawn from the same genre and rated their enjoyment and willingness to pay for one of them. In half of the trials, participants could choose between two options, while in the other half, the choice was made for them by a computer. We hypothesised that having the option to choose would lead to greater enjoyment and increased willingness to pay for books.

### Methods

The University Ethics Committee at Royal Holloway, University of London reviewed and approved this study. All participants provided written informed consent at the start of the experiment.

#### Participants

Forty-nine participants (*M*_age_ = 20.83, *SD* = 1.05, 29 females) were recruited from Royal Holloway, University of London. Our inclusion criteria were native English speakers aged 18 to 24 with normal hearing. We excluded any participants who reported having any neurodevelopmental conditions, neurological disorders, or any speech and language disorders.

#### Choice task

##### Stimuli

Thirty-two extracts from different books were selected using a website that generates book recommendations (www.whichbook.net). Four books were chosen from each of the eight genres (psychological thriller, historical fiction, humour, fantasy, horror, romance, mystery and poetry). These genres were selected based on their popularity from a reading habits survey of 1,500 adults in England (Gleed, 2013).

The selected extracts had a minimum word count of 45 and a maximum word count of 119. Reading ease for each extract was determined using the Flesch Reading Ease scale, which indexes difficulty on a scale of 1 to 100 based on the number of words per sentence and the number of syllables in each word. Lower values indicate more challenging text. Chosen extracts had a minimum reading ease of 60.9 and a maximum reading ease of 100. Stimuli are openly available on the OSF (https://osf.io/fa8sp/).

##### Design

During the task, participants read 32 extracts drawn from unfamiliar books. In half of the trials, participants were able to select which book extract they wanted to read (choice trials) and in the remaining trials an extract was selected for them (no-choice trials). To counterbalance the assignment of extracts to condition (choice/no-choice), extracts were divided into two sets (Set A and Set B). Half of the participants encountered Set A in the choice condition and Set B in the no-choice condition. The other participants received Set B in the choice trials and Set A items in no-choice trials. Additionally, the book ‘selected’ in the no-choice trials was counterbalanced across participants to avoid any biases due to book selection. We consequently had four lists (counterbalancing for sets presented in the choice trials and no-choice trials and counterbalancing for the preselected book in the no-choice trials). This resulted in four counterbalanced stimulus lists; participants were randomly assigned to one of these four lists.

**Figure 1.**
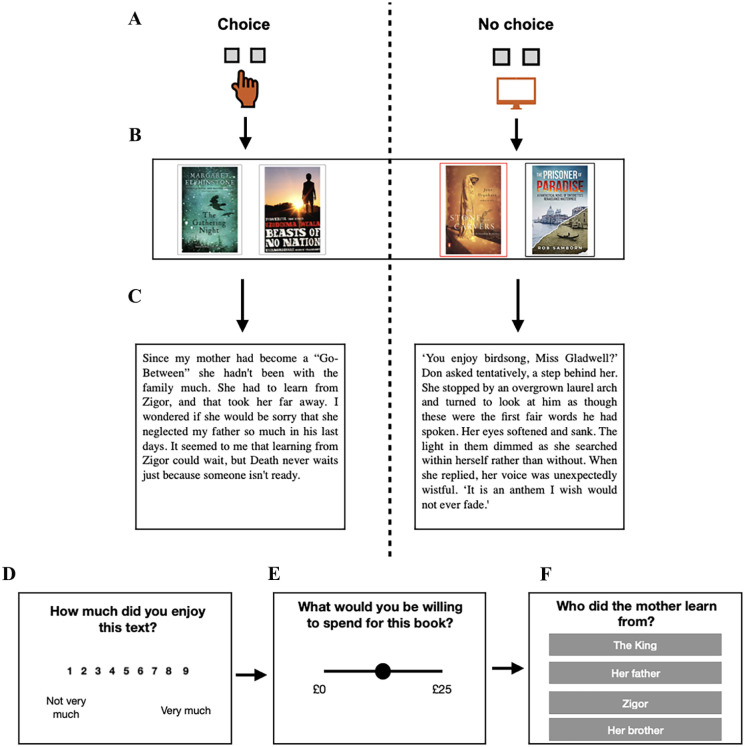
Illustration of the choice task used in Experiment 1. (A) The choice and no-choice cues were displayed at the beginning of each trial. Participants were then shown two book covers (B). In Experiment 2, participants were shown two book genres instead of two book covers (see Supplemental Appendix A for schematic). In the choice condition, participants could pick which book they wanted to read. In the no-choice condition, the book cover with the red outline was selected for them. Participants had 30 s to read an extract from the book (C). They then rated their enjoyment of the extract (D), chose how much they would be willing to pay for the book (E) and answered a question about the text (F).

At the beginning of each trial, a cue was shown to indicate whether the upcoming trial was a choice or a no-choice trial (see Figure 1A). In each trial, participants were shown two book covers (see Figure 1B). After reading each extract, participants reported their enjoyment of each extract on a scale of 1 to 9. They were then asked how much they were willing to pay for each book on a scale from £0 to £25. Participants then answered a multiple-choice question about the text they had encountered; these questions served as attention checks.

We also included a positive control, which is a manipulation allowing us to verify that the experiment is working as expected. As an example, in word learning tasks that examine the effects of training condition on learning, we included a manipulation of word length, as we know longer words are harder to learn (Krishnan et al., 2017, 2018). Here, at the end of the task, participants were also asked to rate their enjoyment of both cues they saw at the beginning of each trial on a scale of 1 to 5. This served as our positive control, as previous studies have shown that participants clearly prefer having a choice, rating the choice cue as more enjoyable than the cue indicating the computer would choose (Leotti & Delgado, 2011; Leotti et al., 2010). This control ensured that participants were paying attention during the task and completing the task as instructed.

#### Procedure

Participants provided informed consent and were invited to complete the experiment online. All tasks were presented on Gorilla.sc (Anwyl-Irvine et al., 2020). Access was restricted to participants using tablets and computers to ensure that the text displayed correctly. Participants were informed that the study would last for 25 min.

First, participants completed the choice task (see Figure 1). They completed 32 trials. On each trial, they saw a cue that indicated whether they would have a choice or not. They then saw two book covers. For trials where they had a choice, they could choose one of the books, and an extract from that book would be displayed for them to read. For trials where they did not have a choice, one of the books would be selected (highlighted in red). They would then see an extract from the selected book. Extracts were displayed for 30 s. After this, participants rated how much they enjoyed the extract, what they were willing to spend on the book, and answered a multiple-choice question about the text.

They then completed a sentence verification task (Garvin & Krishnan, 2022) and the sight word efficiency and phonemic decoding sub-tests from the Test of Word Reading Efficiency (TOWRE-2; Torgesen et al., 2012), which allowed us to assess reading fluency and comprehension.

#### Statistical analyses

We used linear mixed-effects models to analyse the data as they offer several advantages over traditional *t*-tests, including the ability to account for both fixed and random effects, greater flexibility in handling repeated measures and individual variability, and improved statistical power by leveraging all available data (Brown, 2021). All analyses were performed in R (R Core Team, 2020), with linear mixed-effects models created using the lme4 package (Bates et al., 2015). Plots were created using ggprism (Dawson, 2022), gghalves and ggplot2 (Wickham, 2016).

##### Reading enjoyment

We hypothesised that participants would enjoy texts more when they could choose, compared to when a book was selected for them. We therefore fit a mixed-effects linear model with mean-centred enjoyment ratings as the dependent variable. Our fixed effect was choice (choice or no-choice). Random intercepts for item and participant were modelled. We also included by-participant random slopes for choice to account for differences in choice preferences. The maximal model did not converge; so we systematically removed random slopes. Therefore, our final model was:

$$\begin{array}{l} Enjoyment\;\sim \;1\;+\;Choice\;+\;\left(1\;+\;Choice\;|\;Participant\right)\;\\ \;\;\;\;\;\;\;\;\;\;\;\;\;\;\;\;\;\;\;\;\;\;\;\;\;\;\;\;\;\;+\;\left(1\;|\;Book\right) \end{array}$$

##### Willingness to pay

We hypothesised that participants would be willing to pay more for books when they could choose the book they read compared to when a book was selected for them. We fitted a linear mixed-effects model to address this. For this model, willingness to pay was our dependent variable. Our fixed effect was the choice (choice or no-choice). Random intercepts of book and participant were included. We included by-participant random slopes for choice to account for differences in choice preferences. We included random intercepts of participant and book. The maximal model did not converge; therefore, we systematically removed random slopes. Our final model was:

Willingnesstopay~1+Choice+(1|Choice)+(1|Book)

### Results

To ensure that participants understood and performed the task well, we first tested evidence for our positive control. We found that participants reported higher enjoyment for the choice cue (*M* = 3.96, *SD* = 0.96) compared to the no-choice cue (*M* = 3.29, *SD* = 1.22) shown at the beginning of each trial, *t*(90.7) = 3.03, *p* = .003 (see Supplemental Figure 2). This suggested that participants did understand the task and treated the provision of choice as desirable, in line with other decision-making studies (Leotti & Delgado, 2011; Leotti et al., 2010).

**Figure 2.**
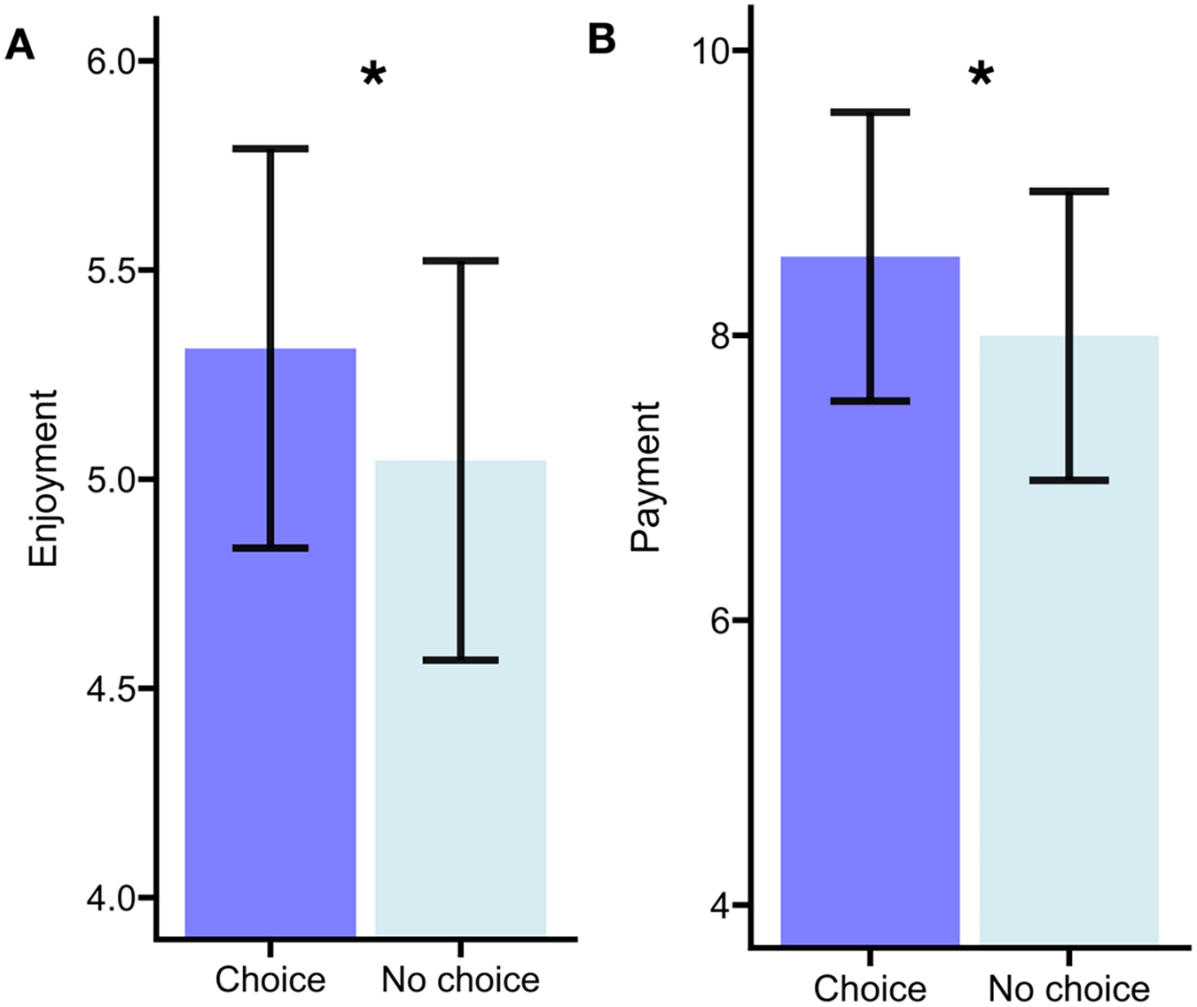
Participants report greater enjoyment (A) and willingness to pay (B) for books they chose (choice condition) in comparison to when a book was selected for them (no choice condition) in Experiment 1. The black lines indicate standard error.

To evaluate evidence for Hypothesis 1, we constructed a mixed-effects model with enjoyment as the dependent variable and choice as a fixed effect. As predicted, choice was associated with increased enjoyment of a book compared to no-choice, β = −.23, *SE* = 0.09, *t* = −2.50, *p* = .016, *d* = 0.36 (see Figure 2A). Participants reported greater enjoyment in the choice condition (*M* = 5.31, *SD* = 1.24) compared to the no-choice condition (*M* = 5.04, *SD* = 1.18).

To evaluate Hypothesis 2, a mixed-effects model with willingness to pay as the dependent variable and choice as a fixed effect was constructed. Choice significantly influenced the willingness to pay, β = −.51, *SE* = 0.17, *t* = −3.07, *p* = .002 (see Figure 2B). Participants were willing to pay more in the choice (*M* = 8.55, *SD* = 1.25) rather than the no-choice conditions (*M* = 7.99, *SD* = 3.33). The effect size was 0.15, indicating this was a smaller effect.

We also tested models (see Supplemental Appendix C) where we modelled the interaction between choice and reading ability (the average of standard scores from the TOWRE sight word efficiency and phonemic decoding subtests). Reading ability was not a significant predictor of enjoyment, β = .009, *SE* = 0.021, *t* = 0.416, *p* = .680, or payment, β = −.054, *SE* = 0.057, *t* = −0.938, *p* = .353. The interaction between choice and reading ability also did not account for significant variance in enjoyment, β = −.017, *SE* = 0.011, *t* = −1.53, *p* = .132, or payment, β = −.014, *SE* = 0.019, *t* = −0.764, *p* = .445. This suggests that the effect of choice was similar across readers with varying abilities.

### Discussion

As predicted, we found that adults reported higher enjoyment and were likely to spend more money on books they chose, compared to when they did not have a choice. This indicates that choice during reading is desirable, and having a choice can increase the value of a selected book. This is consistent with the decision-making literature (Brehm, 1956; Leotti & Delgado, 2011; Leotti et al., 2010; Nanakdewa et al., 2021; Sharot et al., 2009, 2010) and theoretical frameworks of motivation (Guthrie et al., 2005; Hidi & Renninger, 2006; Ryan & Deci, 2000). Although choice is implemented in reading programmes and interventions (Guthrie, Wigfield, et al., 2004; McGeown & Wilkinson, 2021; McGeown et al., 2023), this is the first time the influence of choice has been empirically assessed.

## Experiment 2: providing a choice of genre

In Experiment 1, participants were shown two books from the same genre in each trial. In this experiment, we presented participants with a choice between genres, rather than specific books. Again, we hypothesised that having choice would boost (a) reading enjoyment and be associated with (b) higher monetary valuation, compared to having no choice.

### Methods

#### Preregistration

Having conducted Experiment 1 we preregistered our hypotheses prior to data collection for Experiment 2 (www.osf.io/bhnyg). Using the data from Experiment 1 we conducted a power analysis using the SimR package (Green and MacLeod, 2016) to determine the sample size. The power analysis indicated that for a power of 0.9, with an alpha level of *p* < .05, we needed a sample size of 80.

#### Participants

Eighty participants (*M*_age_ = 21.59, *SD* = 1.57, 38 females) were recruited using prolific.ac. Our inclusion and exclusion criteria were the same as Experiment 1.

#### Procedure

The methods were identical to those in Experiment 1 with one key difference. In the choice trials, participants were given a choice between two genres (see Supplemental Figure 1 in Supplemental Appendix A). Across each trial, participants would see a cue indicating whether they had a choice or no choice. They then saw two boxes with a genre written inside each box. For trials where participants had a choice, they could select a genre and then proceed to read an extract from a book from that genre. For the no-choice trials, a box would be highlighted in red, indicating that the participant would read an extract from a book from that genre. After this, they reported how much they enjoyed reading that extract, and how much they would be willing to pay for a book and answered a multiple-choice question about the extract they had read.

We used the same genres as Experiment 1. They were divided into two sets for generating pairs. Set A included Romance, Horror, Fantasy and Mystery. Set B included Humour, Psychological Thriller, Poetry and History. This resulted in six combinations (Romance and Horror, Romance and Fantasy, Romance and Mystery, Horror and Fantasy, Horror and Mystery and Fantasy and Mystery). Each combination was presented once in the choice condition and once in the no-choice condition (different books were shown in each trial). As before, in the no-choice condition, the selected item was counterbalanced across participants. There were 24 trials in total.

#### Statistical analyses

To analyse the data, we used linear mixed-effects models, following the same protocol as in Experiment 1 and our pre-registered analyses (https://osf.io/fa8sp/). The model structures were identical to those in Experiment 1 and were used to assess the effect of genre choice on (a) reading enjoyment and (b) willingness to pay.

##### Reading enjoyment

$$\begin{array}{l} Enjoyment\;\;\sim \;\;1\;+\;Choice\;+\;\left(1\;+\;Choice\;|\;Participant\right)\;\\ \;\;\;\;\;\;\;\;\;\;\;\;\;\;\;\;\;\;\;\;\;\;\;\;\;+\;\left(1\;|\;Book\right) \end{array}$$

##### Willingness to pay

Willingnesstopay~1+Choice+(1|Choice)+(1|Book)

### Results

As before, enjoyment ratings for the choice cue (*M* = 4.01, *SD* = 0.85) were significantly higher than enjoyment for the no-choice cue (*M* = 3.21, *SD* = 1.03); *t*(79) = 6.96, *p* < .001; see Supplemental Figure 3).

As predicted, and consistent with Experiment 1, having choice increased enjoyment, β = −.19, *SE* = 0.08, *t* = 2.35, *p* = .02 (see Figure 2A). Participants reported higher enjoyment in the choice condition (*M* = 4.51, *SD* = 1.17) compared to the no-choice condition (*M* = 4.32, *SD* = 1.21). This was a small to moderate effect size (Cohen’s *d* = 0.26).

As in Experiment 1, having choice also affected willingness to pay, β = −.40, *SE* = 0.13, *t* = −3.06, *p* = .003 (see Figure 2B). Participants were willing to pay more in the choice condition (*M* = 5.86, *SD* = 3.42) compared to the no-choice condition (*M* = 5.46, *SD* = 3.21). This was a medium-sized effect (Cohen’s *d* = 0.34).

**Figure 3.**
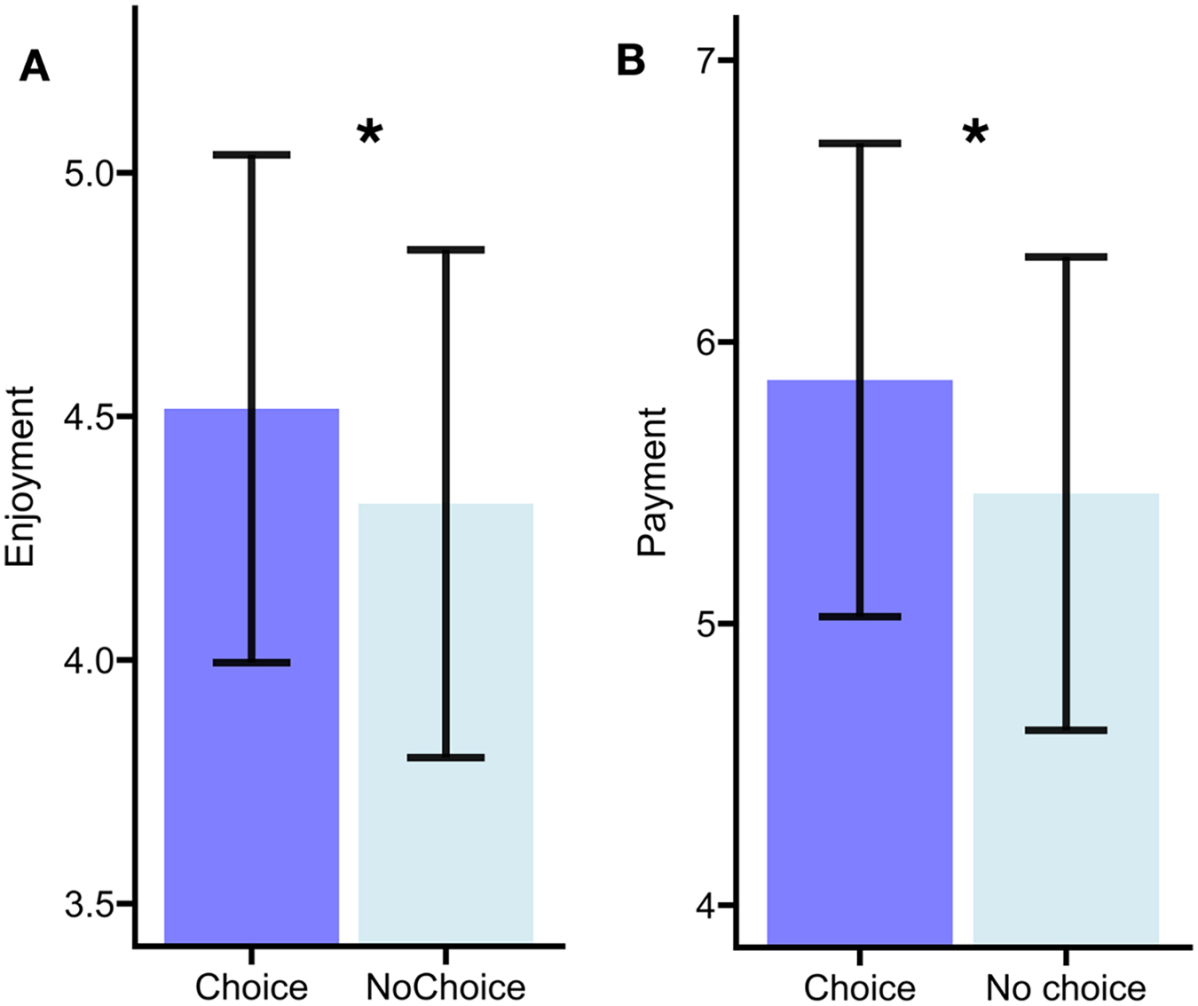
Participants report greater enjoyment (A) and willingness to pay (B) for books they chose in comparison to when a book was selected for them in Experiment 2. The black lines indicate standard error.

Combining data across both studies, we ran exploratory analyses to assess the influence of reading ability on enjoyment and payment. Reading ability was not a significant predictor of enjoyment, β = .003, *SE* = 0.014, *t* = 0.228, *p* = .820 or willingness to pay β = −.054, *SE* = 0.057, *t* = −0.938, *p* = .353. Additionally, the interaction between choice and ability did not account for significant variance in enjoyment, β = −.005, *SE* = 0.009, *t* = −0.608, *p* = .545 or payment, β = −.015, *SE* = 0.019, *t* = −0.764, *p* = .445 (Figure 3).

### Discussion

In line with our predictions, adults reported higher enjoyment for books in the genre that they could choose to read compared to those that were preselected for them. They were also willing to pay more for books from the genre they chose.

## General Discussion

**Figure 4.**
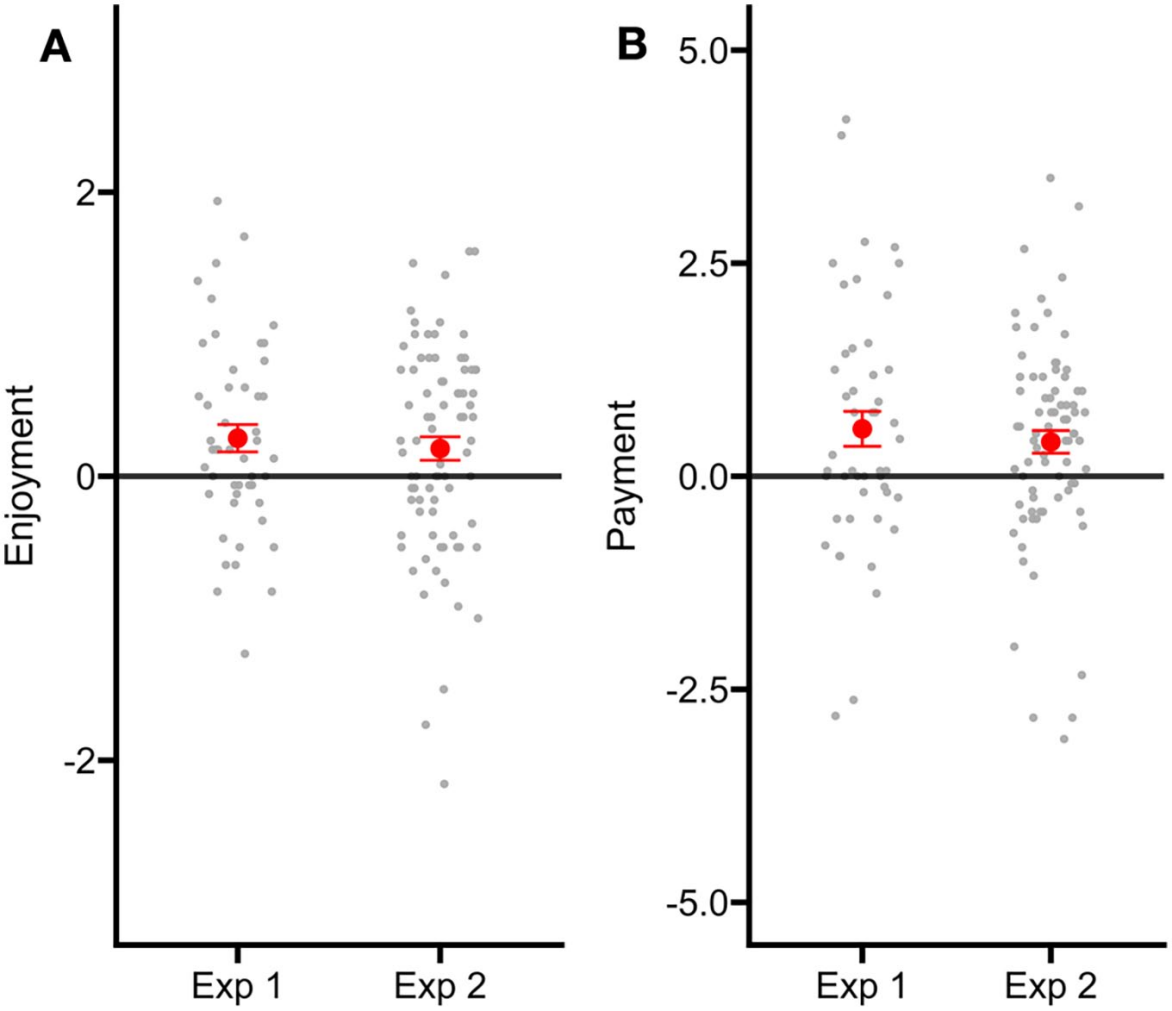
In both Experiment 1 and Experiment 2, there was a similar difference in enjoyment ratings for books when participants selected books in the choice condition (A). Participants were willing to pay more for books when they could choose which book they wanted to read in Experiments 1 and 2 (B).

We investigated how providing choice could affect reading enjoyment and willingness to spend money to buy a book. By adapting our novel reading motivation paradigm (Bains et al., 2023), we were able to establish the value of choice across multiple decisions. In two experiments, we observed that adults reported higher enjoyment for books and were willing to spend more money when they were able to exercise choice. Interestingly, we found that the nature of choice provided – a choice of books or a choice of genre – did not substantially alter our findings (see Figure 4). These findings strongly align with literature suggesting that having choice boosts the subjective value of the chosen stimulus (Brehm, 1956; Leotti & Delgado, 2011; Leotti et al., 2010; Nanakdewa et al., 2021; Sharot et al., 2009, 2010).

Previous studies have largely evaluated the effect of choice on reading by examining autonomy support (Bureau et al. 2022), or through examining motivation within a programme of activities, for example, CORI (Guthrie & Humenick, 2004; Guthrie, Wigfield, et al., 2004; Guthrie et al., 2007; Wigfield et al., 2004, 2008). While these have shed light on the importance of how choice might be leveraged in naturalistic settings, it is challenging to disentangle if it is solely the provision of choice that influences reading motivation. Our robust experimental paradigm allowed us to directly manipulate the influence of choice, averaging across different kinds of books and sampled across genres. Our findings provide strong empirical support for the effect of choice on reading motivation, as indexed by willingness to accept costs. Why do we observe this effect? We have previously highlighted that choice could be a way to express interest or alter preferences due to having a sense of agency. In our study, we had a relatively limited selection of books/genres to minimise the effect of interest, and our careful counterbalancing was designed to mitigate item effects. Further, participants had relatively limited information (i.e. the book cover) upon which to base their choice. Yet, we still find that the act of choice within this limited set of options was associated with increased enjoyment. For this reason, we believe it is more likely that the sense of agency or control conferred through choice drives our effect. In more real-world settings, it is likely that this sense of agency interacts with interest to enhance motivation. For instance, when people are curious about a topic, they are more likely to display motivated behaviour, such as waiting or paying for answers (Garvin & Krishnan, 2022; Kang et al., 2009; Marvin & Shohamy, 2016). Indeed, the Self-Determination Theory posits that interest and autonomy align to make the effect of providing choice stronger (Ryan & Deci, 2000; Ryan et al., 2021).

Across both experiments, adults were also willing to pay more for books they chose to read, regardless of the book itself or genre. In our previous work (Bains et al., 2023), we imposed a temporal cost for reading, and found that participants decided to wait when they enjoyed stimuli greatly. Here, we chose to assess payment as this offered higher face validity and kept the task reasonably short. However, one limitation of this approach is that we did not assess whether participants would spend this money, for example, by operationalising a few of the bids. This was logistically challenging to do in the context of an online study. Yet, we found that participants chose to generally pay the average value of a paperback (between £8.55 and £5.86), with the average gain associated with choice being 65p. This suggests that participants were providing ratings in line with real-world prices. This offered an important line of converging evidence, as enjoyment does not always translate into a willingness to accept costs. We should note a possible limitation in interpreting these data – it is possible that the act of thinking and assessing value to the text can increase its perceived value (Eccles & Wigfield, 2020). Repeatedly asking about value may have contributed to higher overall ratings of enjoyment. We think this is somewhat unlikely, given that participants were in the average range of a paperback. Yet, even if this is the case, this should occur in both the choice and no-choice conditions, and so it would not limit our interpretation of choice. It is highly unlikely that valuation would systematically bias enjoyment solely in the choice condition. However, this could be tested in a future study where no valuation judgement is made.

We evaluated whether the effect of choice differed by reading ability, testing whether better readers were more likely to benefit from the provision of choice by showing greater engagement or motivation. However, we found no evidence for an interaction between reading ability and the effect of choice, suggesting all adult readers benefit from choice.

Previous work has suggested that choice may influence reading comprehension, presumably through increased interest in the material (Bains et al., 2023; Fridkin, 2018; Kakoulidou et al., 2021). In this study, we asked participants one multiple-choice question for each extract as an attention check. These questions were literal and straightforward to answer. Our comprehension data showed ceiling effects; it is therefore unsurprising that we saw no relationships between the provision of choice and comprehension, as our comprehension measure was not sensitive to individual differences (Supplemental Appendix B). Future studies with more sensitive comprehension measures are needed to determine whether choice influences reading comprehension or whether its effects are restricted to intrinsic motivation.

In this study, in each trial, we only had a choice between two books. In future research, it will be important to evaluate how multiple stimuli can affect the value of choice in the reading domain, ideally in naturalistic environments such as libraries and bookstores. Iyengar and Lepper (2000) found that having too much choice decreased participants’ valuation of a stimulus. For example, students were more likely to complete an extra credit assignment when given six rather than 24 topics to choose from. Similarly, they were more likely to purchase jam when given six options rather than 24. Although this runs counter to popular norms suggesting more choice is better, too much information may reduce the value of choice.

Our findings support the inclusion of choice in reading programmes, especially those focused on adults, such as The Reading Ahead programme by The Reading Agency. Indeed, given our data, we argue that providing agency is as important as providing a selection aligned with interest, and this could be easily implemented (e.g. two books in a book giving programme rather than one).

Reading enjoyment declines over the adolescent and teenage years (Clark & Picton, 2021; Clark & Teravainen-Goff, 2020; McGeown & Wilkinson, 2021), and choice may offer a helpful instructional practice. However, as our work focused on adults, generalisability to children and teenagers needs to be specifically evaluated in future studies. In teenagers, we predict that choice is likely to play an even more important role than in adults, as autonomy generally emerges as an important factor in public health interventions (Calvert et al., 2019; Pederson et al., 2016). When evaluating the effect in very young children, costs will need to be measured differently, as waiting or paying may not be developmentally appropriate for this group.

In summary, our studies show that choice can boost reading enjoyment and willingness to spend money on books. This suggests that incorporating a simple element of personal choice could boost reading enjoyment. This has important implications in the design of reading and broader educational interventions.

## Supplemental Material

sj-docx-1-qjp-10.1177_17470218251370916 – Supplemental material for Providing choice enhances reading motivation Supplemental material, sj-docx-1-qjp-10.1177_17470218251370916 for Providing choice enhances reading motivation by Amrita Bains, Carina Spaulding, Jessie Ricketts and Saloni Krishnan in Quarterly Journal of Experimental Psychology
