# Supplementary material for: Providing choice enhances reading motivation
Source: Q J Exp Psychol (Hove). 2025 Aug 18;79(4):959–70. doi: 10.1177/17470218251370916 (PMC12982546; doi:10.1177/17470218251370916)

## **6. Supplementary materials for:**

Providing choice enhances reading motivation

Amrita Bains^1,2^

Carina Spaulding^3^

Jessie Ricketts^3^

Saloni Krishnan^1,4^

^1^ Department of Psychology, Royal Holloway, University of London, Egham Hill, Surrey TW20 0EX, United Kingdom.

^2^ Department of Experimental Psychology, University of Oxford, Anna Watts Building, Woodstock Rd, Oxford OX2 6GG.

^3^ The Reading Agency, 24 Bedford Row, London, WC1R 4EH, United Kingdom.

^4^ Division of Psychology and Language Sciences, University College London, 2 Wakefield St, London WC1N 1PF.

Corresponding authors:

Amrita Bains <amrita.bains@sjc.ox.ac.uk>

Saloni Krishnan <[s.krishnan@ucl.ac.uk](mailto:s.krishnan@ucl.ac.uk) >

**Appendix A
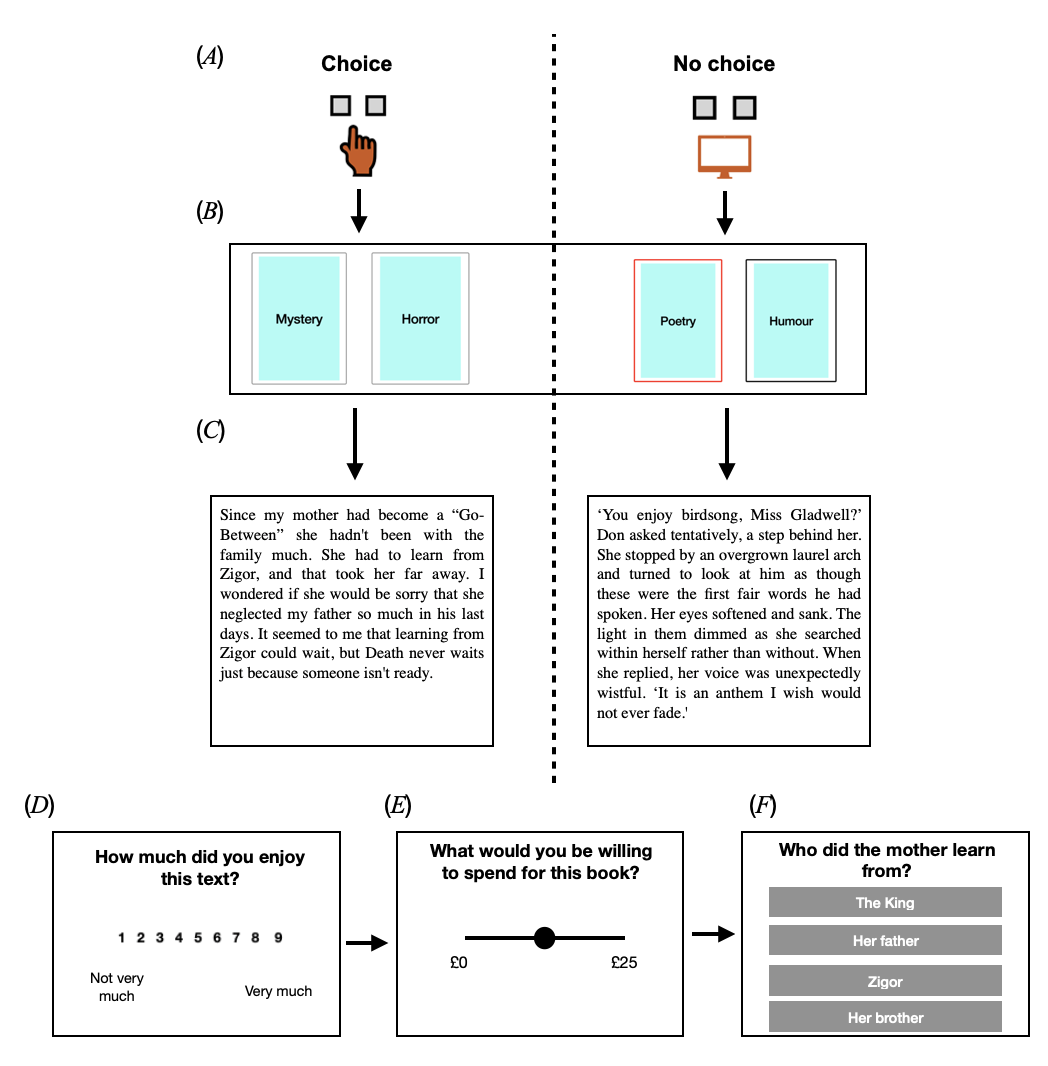
**

*Supplementary Figure 1****.*** The choice task for Experiment 2. Panel (A) shows the choice and no-choice cues presented at the start of each trial. Participants were then shown two genres (B). In choice trials, they selected the genre of the book they wanted to read. In no-choice trials, the selected genre was highlighted in red. Next, they read an extract from a book in that genre (C), rated their enjoyment of the extract (D), indicated how much they would be willing to pay for the book (E), and answered a comprehension question (F).

**Appendix B**

**
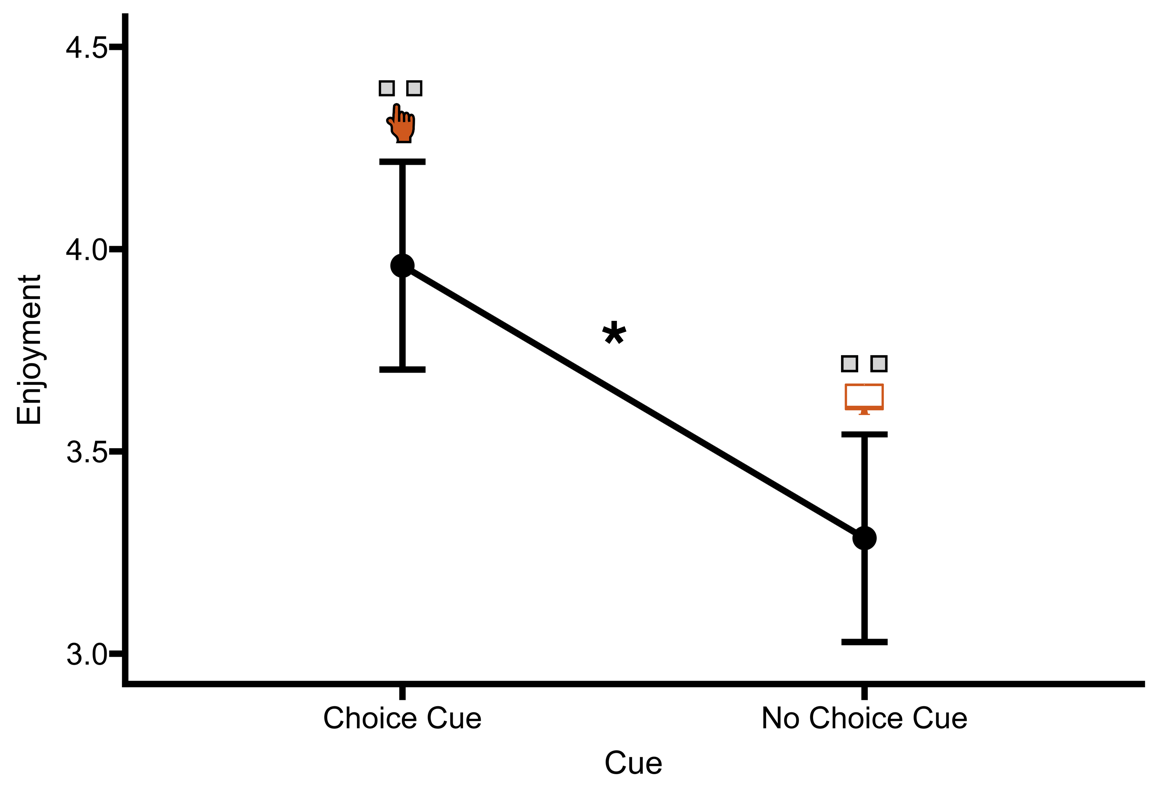
**

*Supplementary Figure 2.* In Experiment 1, participants rated the choice cue as more enjoyable compared to the no choice cue.

**
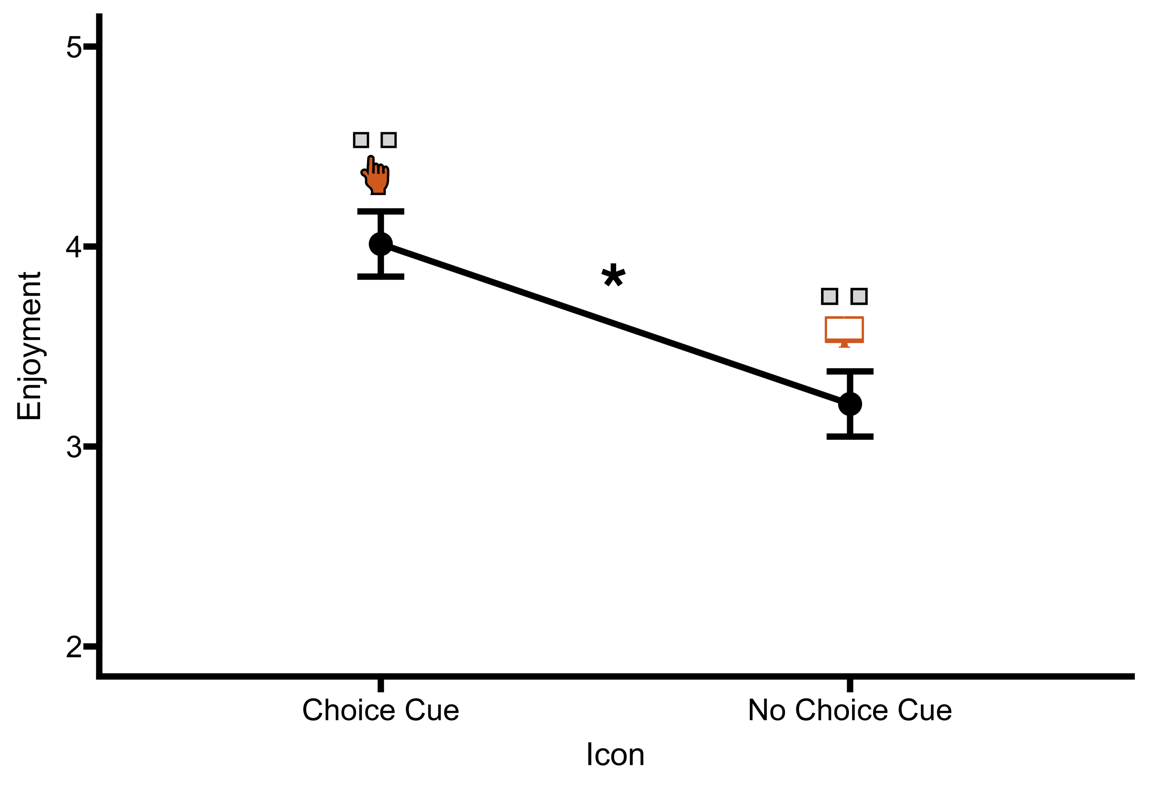
**

*Supplementary Figure 3.* In Experiment 2, participants rated the choice cue as more enjoyable compared to the no choice cue.

**Appendix C**

**Influence of choice on reading comprehension**

In our study, participants answered one comprehension question for each book they read. We investigated whether there was an effect of choice versus no choice on reading comprehension. We constructed mixed effects logistic models for both experiments with comprehension as the dependent variable and choice as the fixed effect. Random intercepts of participant and book were included. We found no significant effect of choice on comprehension in Experiment 1, β=.27, SE=.04, *z*=1.93, *p*=0.053, and in Experiment 2, β=-.10, SE=.15, *z*=-.68, *p*=0.50.

*Supplementary Figure 1*. Distribution of comprehension accuracy in Experiment 1 (panel A) and Experiment 2 (panel B).


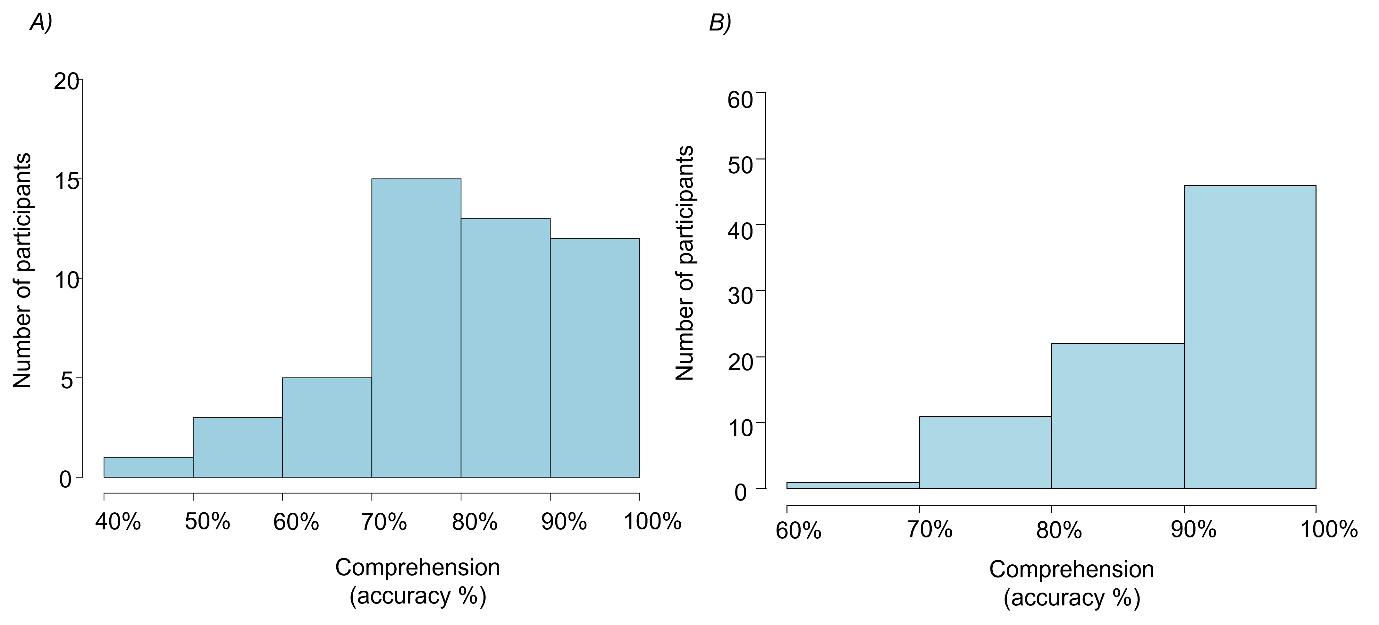


*Supplementary Figure 3.* Distribution of reading ability scores as measured by the sentence verification task and TOWRE subtests; sight word efficiency and phonemic decoding in Experiment 1.


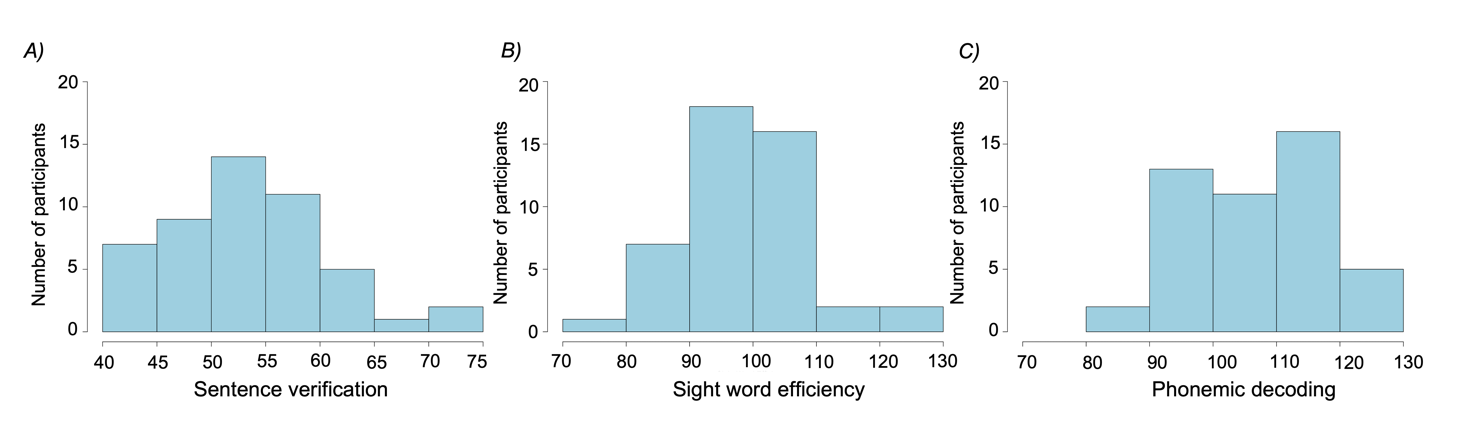


*Supplementary Figure 3.* Distribution of reading ability scores as measured by the sentence verification task and TOWRE subtests; sight word efficiency and phonemic decoding in Experiment 2.


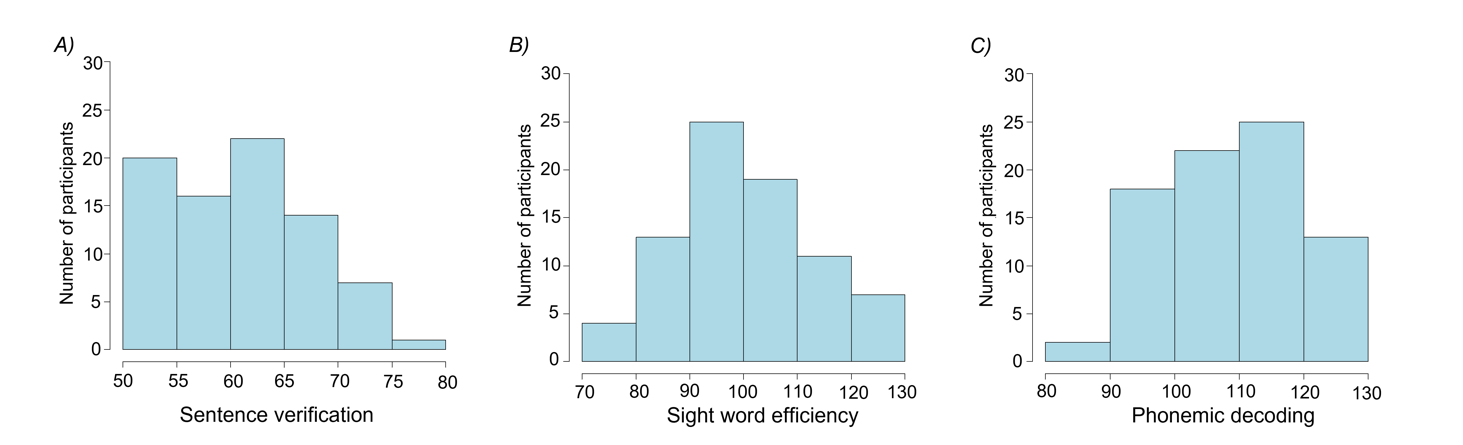


Supplementary Figure 4. Trials for each participant were split into two groups (trials labelled with an odd number or an even number) and correlated to assess internal reliability for our measures of reading enjoyment and willingness to pay. Data points for Experiment 1 are shown in Panel A, and data points for Experiment 2 are shown in Panel B.


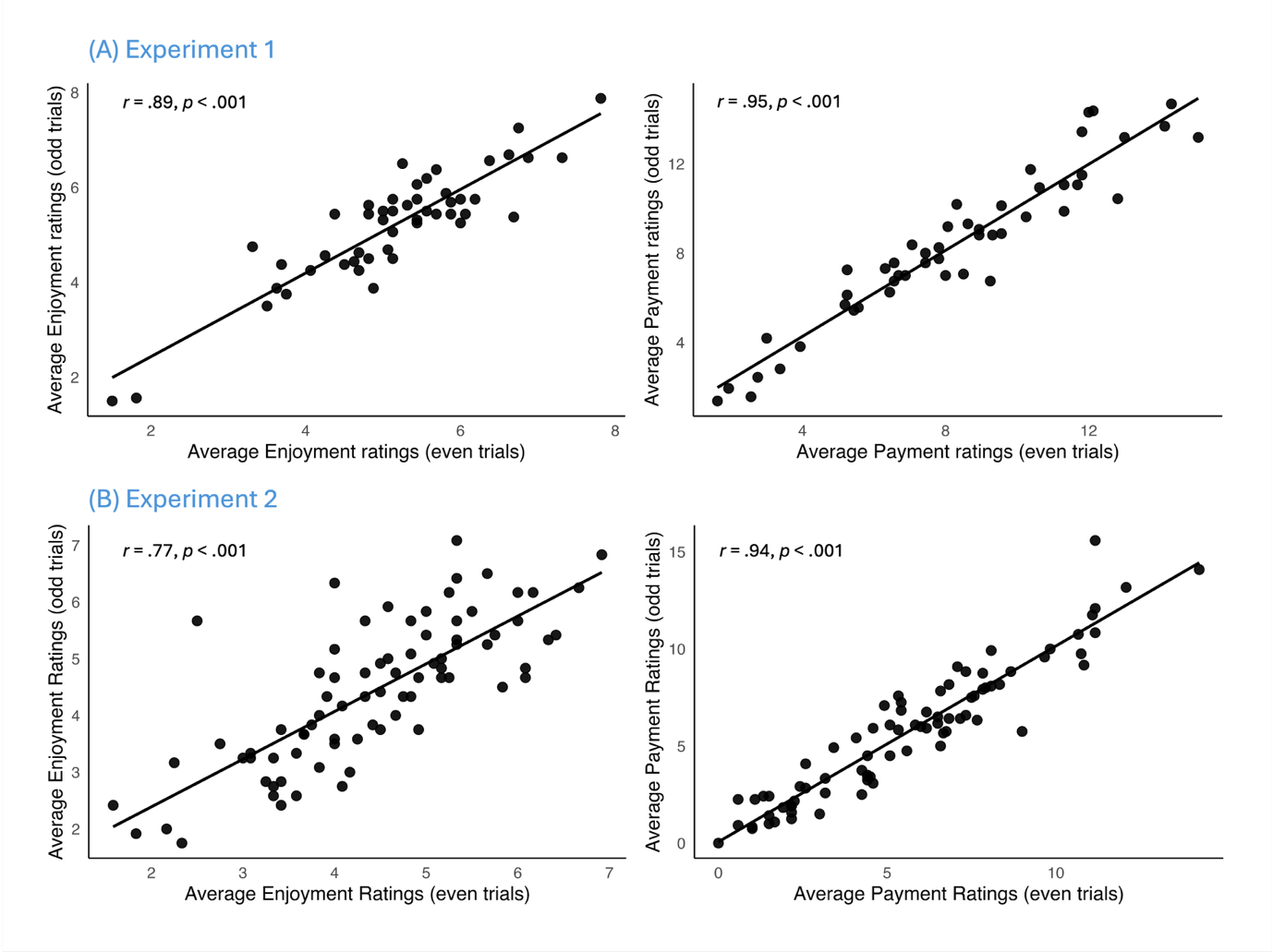

Supplement: sj-docx-1-qjp-10.1177_17470218251370916 – Supplemental material for Providing choice enhances reading motivation [file sj-docx-1-qjp-10.1177_17470218251370916.docx]
